# Some General Aspects of Poisons—III

**Published:** 1909-08-28

**Authors:** 


### Medico-Legal Points.

### SOME GENERAL ASPECTS OF POISONS—III.

### Ammonia.

The vapour of strong ammonia is poisonous. It may destroy life by producing violent inflammation of the larynx and of the lungs. The vapour produces a feeling of choking, with a sense of great heat in the throat: it appears to suspend the power of breathing, and the pain and heat in the throat remain for a long time. Solution of ammonia applied to the skin acts as a corrosive, and may inflame or cause the destruction of the parts which it touches. At the Stafford Summer Assizes, 1873 (R. *v.* Gavan), a man was convicted of throwing an ammoniacal liquid over the prosecutrix with intent to injure her. It was a liniment containing a strong solution of ammonia. The liquid was thrown in her face, and some portion reached the eyes; but she recovered from the effects. A weak solution acts as an irritant to the skin, while a strong solution causes vesication and destruction of the part. A very interesting report is given by Dr. Fairbrother (*St. Louis Med. and Surg. Jour.* 1887, vol. lii., p. 272), where three men lost their lives and a fourth was permanently injured by an accident when setting up an ammonia ice-machine. They were exposed to the gas for some three minutes. One when dragged out was comatose and unconscious, and died in fifteen minutes. Another was suffering as though from chloroform in the stage of excitement—*i.e.* he was unconscious in wild delirium, and could not stand. He was not improved by the injection of half a grain of morphine sulphate, and died suddenly in two hours. The third was entirely conscious and walked home. He could swallow readily, and talk easily, but complained of occasional difficult breathing, and after five hours' time, in a sudden attack of dyspnœa, he gave two or three gasps and died. The last suffered from bronchial irritation for some months. His leg was broken by the fall and had to be amputated, and he became partially paralysed on one side.

### Sulphuric Acid.

This substance was known to the alchemists, and has been an article of commerce since the Middle Ages under the common name of oil of vitriol. The term oil of vitriol is strictly applied only to sulphuric acid, which has an oily consistency and a great specific gravity (from 1.800 to 1.845). It was so called because it was obtained by the distillation of green vitriol, of which it was considered to be the oil or spirit. It is in this state eminently corrosive, and this corrosive property is lost when the oily consistency is removed by dilution with its bulk of water. Sulphuric acid is frequently taken as a poison by suicides, but probably there is no case in which the sufferings of a person before death are more intense. Sulphuric acid, in common with the other mineral acids, is not often used for murder, the taste betraying the attempt to the victim, and the symptoms of death from it being characteristic. Cases, however, have been occasionally reported where it has been purposely administered to children, and also to intoxicated adults, with fatal results. It is not uncommonly used for suicide, especially among the lower classes on the Continent. This strong acid is not infrequently used for throwing upon the face and hands of rivals or enemies. This practice is largely confined to the gentler sex, and often results in terrible and permanent disfigurement, and sometimes a very serious injury to the health.

### Antimony.

Tartar emetic, which is so largely used in medicines, has given rise to most of the cases of antimony poisoning. It is a white crystalline solid readily soluble in water. It may cause both acute and chronic poisoning, some of the cases of criminal poisoning recorded being of the latter variety. Criminal poisoners rarely resort to one big dose of antimony for their purposes; they more commonly give small doses at intervals. The principal symptoms produced by tartar emetic given at intervals in small doses to healthy persons are those of gastro-intestinal catarrh, namely nausea and vomiting of mucous and bilious liquids; great depression; watery purging, followed often by constipation of the bowels; small, contracted, and frequent pulse; loss of voice and muscular strength; coldness of the skin, with clammy perspiration; and death from exhaustion. Several cases have occurred which show that tartar emetic has been thus criminally employed. In addition to the cases of Ann Palmer and J. P. Cook, there are those of R. *v.* M'Muller (Liverpool Summer Assizes, 1856); R. *v.* Freeman (Drogheda Spring Assizes, 1857), and the cases of the James family at Liverpool (R. *v.* Winslow, 1860, 8 Cox, C.C., 397). The prisoner Winslow was indicted for the murder of his mistress, Ann James. It was clearly proved that antimony had been administered to the deceased not only from the symptoms, but by the detection of the poison in the urine during life. The deceased was at the time labouring under malignant disease affecting the cæcum and stomach, but it was alleged that her death had been accelerated by antimony. The prisoner was acquitted, owing to the difficulty of proving the act of administration. The poison had been given at intervals in small doses, and as deceased survived about a fortnight after the last dose, it was found only in traces in the various organs. The death of this woman led to the exhumation of the bodies of three of her relatives who had lived in the same house with her and the prisoner, and had died some months previously under suspicious circumstances. The viscera of these bodies were examined, and in each case antimony was found in small quantity, but still extensively diffused through the organs. So far as the history of their cases could be obtained, they were victims of chronic poisoning by antimony. This cause of death was not suspected at the time.

It must be remembered that the discovery of antimony in the contents of a stomach is by no means a proof of its having been taken or administered as a poison, since tartar emetic is frequently prescribed as a medicine, and often taken as such by persons of their own accord. The presence of any quantity, if not lawfully administered as a medicine, is always a suspicious fact, and demands explanation. In two cases of criminal administration in small doses the quantity found in each body did not exceed three grains. The detection of antimony in the tissues does not necessarily indicate that it has been criminally administered nor that it has caused death: but its presence should be reasonably accounted for, as antimony may have been unlawfully administered. In several cases of suspected death from poison, deposits on copper, evidently of an antimonial nature, have been obtained from the liver or tissues. On inquiry it has been found that antimonial medicines had been taken shortly before death. Conversely, when no antimony is found, or the metal is present in the tissues in minute quantity, it is still consistent with medical experience and observation that the person may have died from antimony.

An extraordinary trial for murder by alleged poisoning with this substance took place at Annapolis, U.S.A., in 1881. Mrs. Wharton was charged with poisoning her friend General Ketchum. The trial lasted fifty-two days, and a huge amount of scientific evidence was brought forward for the prosecution and defence, apparently owing to the high social position of the parties. The General died after a short illness, but the symptoms, taken as a whole, bore no resemblance to those observed in poisoning with antimony, although poisoning was suspected during life. The appearances in the body proved nothing for or against antimonial poisoning, and some physicians of experience deposed that the symptoms and appearances were consistent with disease affecting the membranes of the brain and spinal marrow. On examining the chemical evidence, it appears that sulphide of ammonium alone was employed for the detection of antimony, and a red-brown sulphide resembling that of antimony was obtained; but the quantity obtained as sulphide was only four-tenths of a grain, estimated as equivalent to eight-tenths of a grain of tartar emetic. Thus the chemical analysis brought out only a fraction of a grain, not amounting to one-twentieth part of the quantity said to be present; and no separation of antimony in the metallic state was made to corroborate the inference drawn from the coloured precipitate produced by sulphuretted hydrogen. No chemical results were produced in court, although 20 grains would have allowed of the production of metallic antimony in a few minutes by copper, tin, zinc, and platinum, or by Marsh's process. The evidence that antimony was really there was not satisfactory, and that 20 grains were present in the stomach was wholly unproved. The chemical evidence does not therefore conflict with the pathological evidence, for it failed to show with clearness and distinctness the presence and proportion of the poison said to have been found. The prisoner was acquitted. (*Amer. Jour. Med. Sc.*, April 1872, p. 329.)

### Salts of Copper.

Acute poisoning by salts of copper is not common. It is seldom that these poisons are designedly administered with homicidal intentions, since their detection, both by the colour and taste is easy. A husband attempted to poison his wife by adding verdigris to a dish of beans. The bad taste prevented her from eating them. He buried the cooked mess in his garden, from which it was disinterred, and then examined by chemists. They proved the certain presence of the metal. He was condemned to hard labour for life (*Jour. de Chimie*, *Chevalier*, 1854). Cases of poisoning from these salts may, then, be divided into those in which a large dose is swallowed, either by accident or with a view to suicide, and those which proceed from the contamination of food by unclean copper vessels, or by the salts of copper used as colouring matters for confectionery, etc., the injurious qualities of which will depend upon the amount of contamination. If metallic copper is subjected to the action of water, acetic acid or vinegar, or hydrochloric acid, it is not affected unless the fluid is in contact with air or oxygen; so that copper vessels can be used for cooking if the fluid be removed from the copper vessel while it is hot; in which case the vinegar, fatty acids, or water will have no effect upon the copper; but if the fluids be allowed to stand in the copper vessel for several hours when cold, air is absorbed, the copper is in part oxidised, dissolved by the acid, and impregnates the food.

### Hydrocyanic Acid and its Salts.

Hydrocyanic acid is a transparent, colourless, volatile liquid, with a pungent, acrid taste, which latter may be easily concealed in its administration in medicine or alcoholic beverages. It has a peculiar odour, often likened to that of bitter almonds, or the kernels of peach stones. Hydrocyanic acid may be obtained from many vegetables, as from bitter almonds, apple pips, the kernels of peaches, apricots, cherries, plums, and the root of mountain ash. Prussic acid does not exist ready formed in these plants, but is the result of the reaction of water upon amygdalen in the presence of a ferment emulsion. Hence, if any of the above substances are found in the stomach, the question may arise whether the indications of the presence of prussic acid are due to their decomposition or to the acid swallowed as such. The only manner in which doubt, arising from this circumstance, can be satisfied, is the obtaining by chemical analysis of a larger quantity of the acid from the contents of the stomach than these substances could yield.

